# Supplementary material for: Mapping Genetic Variation in Arabidopsis in Response to Plant Growth-Promoting Bacterium Azoarcus olearius DQS-4T
Source: Microorganisms. 2023 Jan 28;11(2):331. doi: 10.3390/microorganisms11020331 (PMC9961961; doi:10.3390/microorganisms11020331)
Supplement: Supplementary file 1 [file microorganisms-11-00331-s001.zip › microorganisms-2068940-supplementary.pdf]

microorganisms

MDPI

Supplementary Material

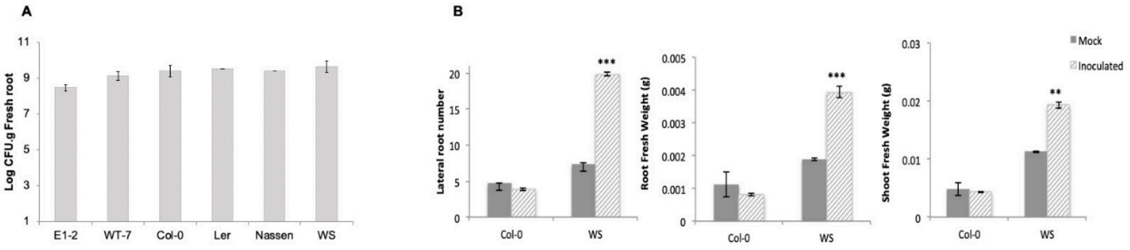

Figure S1. Differential growth responses of Arabidopsis accessions to the beneficial bacterium *A. olearius* DQS-4^T. A. Bacterial cells recovered from roots of Col-0 and WS five days after treatment with DQS-4^T. No bacterial cells were recovered from the mock treatment. Results are shown in log of colony forming units (CFU) per gram of fresh root tissue. Bars are the average of root samples per treatment (n=15). B. Growth parameters analyzed 10 days after DQS-4T treatment in Col-0 and WS accessions. Bars are average of samples (n=32). Statistical significance is represented by ** p>0.01, ***0.001 calculated by one-way Anova.

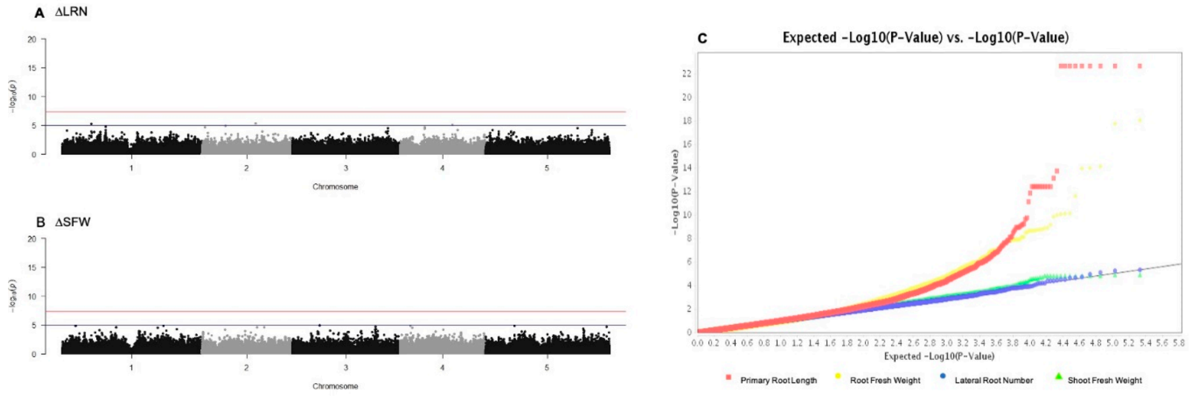

Figure S2. Manhattan plot representing SNPs upon GWA mapping. A. Lateral root number (LRN) and B. Shoot fresh weight (SFW), no significant SNPs were found at a threshold $-\log_{10}$ (P) = 10^{-7} for both traits. C. Q-Q plot representation of data distribution within 305 Arabidopsis accessions. Each color represents a different set of data in $-\log_{10}$ (*p-value*), red = PRL, yellow = RFW, blue = LRN and green = SFW. According to Q-Q plot PRL and RFW showed high variability in distribution within the data, unlike LRN and SFW.

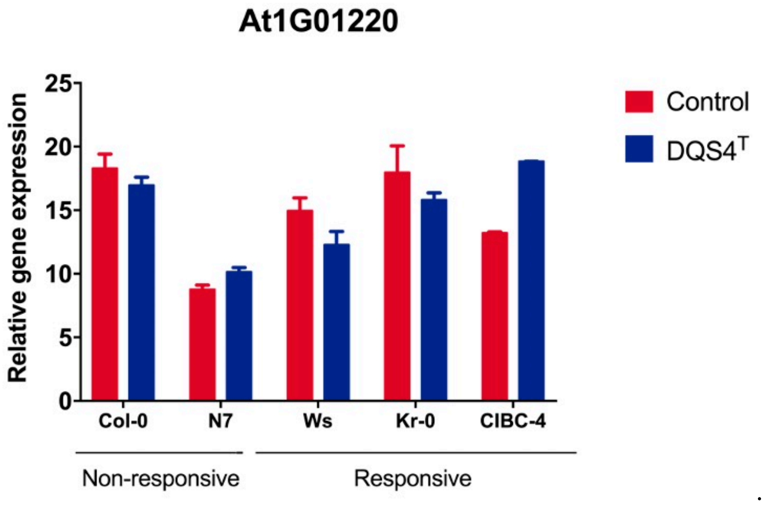

Figure S3. Relative gene expression of At1G01220, L-fucose gene. RT-qPCR analysis of mRNA expression from root tissue of non-responsive genotypes (Col-0 and N7) and responsive genotypes

(Ws, Kr-0 and CIBC4) 7 days after inoculation. The values are expressed as the relative quantity with respect to the control. The data represent the average of three independent experiments.

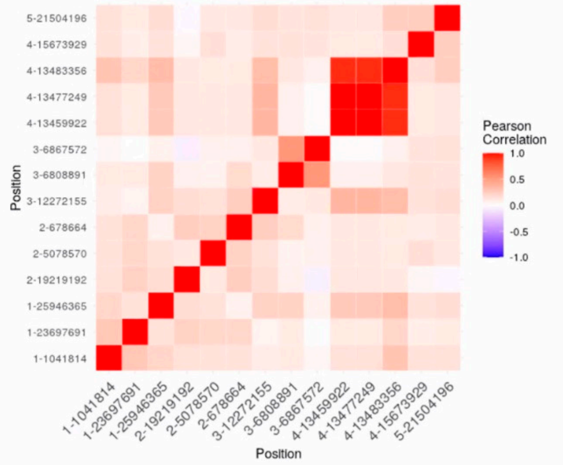

SNPs	Inclusion Prob.	MAF
2-678664	0.0877	0.0272109
2-5078570	0.349	0.0272109
2-19219192	0.02279	0.0238095
3-6808891	0.08144	0.0238095
3-12272155	0.000197	0.0238095
4-13459922	0.012232	0.0238095
4-13477249	0.02186	0.0238095
4-1383356	0.3312	0.0272109
5-21504196	0.0936	0.0238095

Figure S4. Pearson's correlation matrix 10 kb region surrounds a highly significant SNP 13459922 at chromosome 4. SNPs in LD with 13459922 are shown in the table with SNPs physical location.

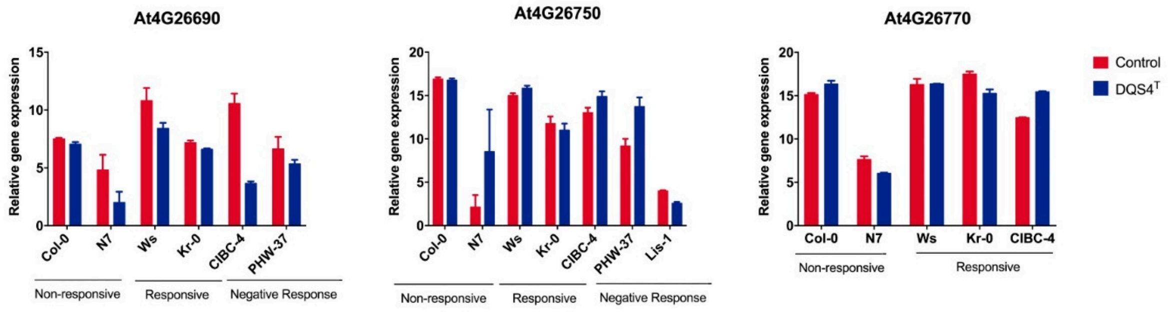

Figure S5. Relative gene expression of At4G26690, At4G26750 and At4G26770. RT-qPCR analysis of mRNA expression from root tissue of non-responsive genotypes (Col-0 and N7), responsive (Ws, Kr-0 and CIBC4) and negative response (PHW-37 and Lis-1) 7 days after inoculation. Lis-1 showed no expression detected in At4G26690 and AtG426770 genes. The values are expressed as the relative quantity with respect to the control. The data represent the average of three independent experiments.
